# Pre-Power-Stroke Cross-Bridges Contribute to Force Transients during Imposed Shortening in Isolated Muscle Fibers

**DOI:** 10.1371/journal.pone.0029356

**Published:** 2012-01-05

**Authors:** Fabio C. Minozzo, Lennart Hilbert, Dilson E. Rassier

**Affiliations:** 1 Department of Kinesiology and Physical Education, McGill University, Montreal, Quebec, Canada; 2 Department of Physiology, McGill University, Montreal, Quebec, Canada; 3 Centre for Applied Mathematics in Biosciences and Medicine, McGill University, Montreal, Quebec, Canada; 4 Meakins-Christie Laboratories, McGill University, Montreal, Quebec, Canada; 5 Department of Physics, McGill University, Montreal, Quebec, Canada; Institute of Enzymology of the Hungarian Academy of Science, Hungary

## Abstract

When skeletal muscles are activated and mechanically shortened, the force that is produced by the muscle fibers decreases in two phases, marked by two changes in slope (P_1_ and P_2_) that happen at specific lengths (L_1_ and L_2_). We tested the hypothesis that these force transients are determined by the amount of myosin cross-bridges attached to actin and by changes in cross-bridge strain due to a changing fraction of cross-bridges in the pre-power-stroke state. Three separate experiments were performed, using skinned muscle fibers that were isolated and subsequently (i) activated at different Ca^2+^ concentrations (pCa^2+^ 4.5, 5.0, 5.5, 6.0) (n = 13), (ii) activated in the presence of blebbistatin (n = 16), and (iii) activated in the presence of blebbistatin at varying velocities (n = 5). In all experiments, a ramp shortening was imposed (amplitude 10%L_o_, velocity 1 L_o_•sarcomere length (SL)•s^−1^), from an initial SL of 2.5 µm (except by the third group, in which velocities ranged from 0.125 to 2.0 L_o_•s^−1^). The values of P_1_, P_2_, L_1_, and L_2_ did not change with Ca^2+^ concentrations. Blebbistatin decreased P_1_, and it did not alter P_2_, L_1_, and L_2_. We developed a mathematical cross-bridge model comprising a load-dependent power-stroke transition and a pre-power-stroke cross-bridge state. The P_1_ and P_2_ critical points as well as the critical lengths L_1_ and L_2_ were explained qualitatively by the model, and the effects of blebbistatin inhibition on P_1_ were also predicted. Furthermore, the results of the model suggest that the mechanism by which blebbistatin inhibits force is by interfering with the closing of the myosin upper binding cleft, biasing cross-bridges into a pre-power-stroke state.

## Introduction

A long-standing scientific challenge resides in the explanation of how characteristics of the molecular actin-myosin interaction give rise to macroscopically observed phenomena in striated muscles, and how conditions imposed on macroscopic scales affect actin-myosin kinetics. In early experiments to connect macroscopic muscle mechanics to load-dependent cross-bridge kinetics, step shortenings were imposed to fully activated fibers isolated from amphibian muscles [1]. The force transients could be described in four phases: 1) during the fast shortening step, there was a force decrease proportional to the shortening amplitude, 2) during the next 3–5 ms there was a rapid force recovery, 3) during the next 10–50 ms there was an extreme reduction of force recovery, and 4) during the remainder of response, there was an asymptotic recovery towards maximum isometric force. At the end of phase 1, a maximal drop in force (T_1_) was observed and the beginning of phase 2 indicated a transition into an increase of force. A following inflection or even a low peak in the force time course at force (T_2_) indicated the transition into phase 3 [1].

Length ramps performed at constant velocities are now commonly used for studying the molecular mechanisms of muscle contraction [1]–[5], and show force responses that are qualitatively similar to early studies that used step shortening: 1) the force decreases in proportion to shortening, 2) the force decrease becomes less rapid, 3) the force decrease becomes even slower, 4) and the force shows an asymptotic approach to a lowered but constant steady state. Some of these studies show a transition in the force trace from phase 1 to phase 2 (hereafter called critical point P_1_) that occurs at a critical sarcomere length (L_1_), and a transition in the force trace from phase 2 to phase 3 (hereafter called critical point P_2_), that occurs at a critical sarcomere length (L_2_). While phase 1 in force traces is commonly associated with a purely elastic response, the behaviour during phase 2 is attributed to a repartitioning of cross-bridges from the pre to the post-power stroke state, due to an acceleration of the power-stroke step under conditions of lowered mechanical load on myosin cycling [1], [3], [5], [6].

In this study, we reinvestigated the mechanisms responsible for the force transients during a shortening ramp. We examined fibers at different levels of Ca^2+^ activation, and fibers treated with the highly specific myosin inhibitor blebbistatin, which biases cross-bridges into a pre-power-stroke state [7], [8]. Different Ca^2+^ concentrations allowed us to examine the influence of the number of strongly-bound cross-bridges on the force transients during shortening, while blebbistatin allowed us to investigate the effects of cross-bridge partitioning into pre and post-power-stroke states before ramp shortening. While changes in Ca^2+^ concentration did not significantly alter the P_1_ and the P_2_ transitions during shortening, blebbistatin decreased P_1_ significantly during shortening.

We developed a mathematical cross-bridge model with a load-dependent power-stroke transition between pre and post-power-stroke cross-bridge states, which was based on general protein motor kinetics formalisms [9] and single myosin experiments [10]. Three cross-bridge kinetic states were derived in accordance with well-defined biochemical pathways [9], [11], [12] and compatible with current structural models of myosin [13]. Similar to what other models investigating ramp stretches have suggested [6], [14]–[17], pre-power-stroke cross-bridges were found to be a major determinant of the force transients during shortening. The model explained qualitatively the force transients (P_1_ and P_2_) and the lengths at which they happen (L_1_ and L_2_, respectively) observed in our experiments using different shortening velocities. It also predicted the effects of blebbistatin inhibition on P_1_, further indicating that the mechanism by which blebbistatin inhibits active force generation is by preventing the closing of the myosin binding cleft, effectively biasing cross-bridges into a pre-power-stroke state.

## Methods

### Muscle fiber preparation

Small muscle bundles of the New Zealand White rabbit psoas were dissected, tied to wood sticks, and chemically permeabilized following standard procedures [16], [18]. The muscles were incubated in rigor solution (pH = 7.0) for approximately 4 hours, after which they were transferred to a rigor∶glycerol (50∶50) solution for 15 hours. The samples were placed in a new rigor∶glycerol (50∶50) solution with the addition of a mixture of protease inhibitors (Roche Diagnostics, USA) and stored in a freezer (−20°C) for at least seven days. On the day of the experiment, a muscle sample was transferred to a fresh rigor solution and stored in the fridge for one hour before use. A small section of the sample was extracted (∼4 mm in length), and single fibers were dissected in a relaxing solution (pH = 7.0). The fibers were fixed at their ends with T-shaped clips made of aluminum foil, and were transferred to a temperature controlled experimental chamber to be attached between a force transducer (Model 400A, Aurora Scientific, Toronto, Canada) and a length controller (Model 312B, Aurora Scientific, Toronto, Canada). The protocol was approved by the McGill University Animal Care Committee (protocol #5227, valid 2006–2016) and complied with the guidelines of the Canadian Council on Animal Care.

### Solutions

The rigor solution (pH 7.0) was composed of (in mM): 50 Tris, 100 NaCl, 2 KCl, 2 MgCl_2_, and 10 EGTA. The relaxing solution (pH 7.0) used for muscle dissection was composed of (in mM): 100 KCl, 2 EGTA, 20 imidazole, 4 ATP and 7 MgCl_2_. The solutions with pCa^2+^ of 4.5, 5.0, 5.5 and 6.0 (pH 7.0) were composed of (in mM): 20 imidazole, 14.5 creatine phosphate, 7 EGTA, 4 MgATP, 1 free Mg^2+^. These solutions had free Ca^2+^ ranging from 1 nM (pCa^2+^ 9.0 relaxing) to 32 µM (pCa^2+^ 4.5 maximum activation), and KCl to adjust the ionic strength to 180 mM. A pre-activation solution (pH 7.0, pCa^2+^ 9.0) was used before activating the fibers, composed of (in mM): 68 KCl, 0.5 EGTA, 20 Imidazole, 14.5 PCr, 4.83 ATP, 0.00137 CaCl_2_, 5.41 MgCl_2_ and 6.5 HDTA (pH 7.0, pCa^2+^ 9.0). The final concentrations of each metal-ligand complex were calculated using a computer program [19] which takes into account the reaction between the buffers when forming chemical complexes to calculate the final free ionic concentrations.

The solutions containing blebbistatin were prepared according to the following procedures. 1 µL of blebbistatin (Sigma, USA) prepared at 20 mM, previously dissolved in dimethylformamide (DMF), was diluted in 4 mL of activating (pCa^2+^ 4.5) or relaxing (pCa^2+^ 9.0) solutions to reach a final concentration of 5 µM. Care was taken to limit blebbistatin exposure to light, as it loses its effectiveness in wavelengths between 365 nm and 490 nm [20]. A red filter (650 nm) placed on the light source of the microscope was used to avoid exposure when the use of light was necessary during the experiments. Solutions were prepared with both the active and the inactive (+/+) isomers of blebbistatin, which was used as a negative control.

### Experimental protocol

After the fibers were set in the experimental chamber, the average sarcomere length (SL) was calculated in relaxing solution using a high-speed video system (HVSL, Aurora Scientific 901A, Toronto, Canada). Images from a selected region of the fibers were collected at 1000–1500 frames•sec^−1^, and the SL was calculated by fast fourier transform (FFT) analysis based on the striation spacing produced by dark and light bands of myosin and actin, respectively. The fiber diameter and length were measured using a CCD camera (Go-3, QImaging, USA; pixel size: 3.2 µm×3.2 µm), and the cross-sectional area was estimated assuming circular symmetry.

Three separate sets of experiments were performed during this study, using (i) fibers activated at different Ca^2+^ concentrations and shortened at 1 L_o_•SL•s^−1^ (n = 13), (ii) fibers activated in the presence or absence of blebbistatin and shortened at 1 L_o_•SL•s^−1^ (n = 16), and (iii) fibers activated in pCa^2+^ 4.5 and pCa^2+^ 6.0 in the presence of blebbistatin, shortened at varying velocities ranging from 0.125 L_o_•SL•s^−1^ to 2.0 L_o_•SL•s^−1^ (n = 5). All experiments were performed at 5°C.

At the beginning of the experiments, the initial SL was adjusted to 2.5 µm (optimal length, L_o_) before activation. (i) For the experiments with different Ca^2+^ concentrations, fibers were activated at pCa^2+^ of 4.5, 5.0, 5.5 and 6.0 (random order). When force was fully developed in each pCa^2+^, a ramp shortening of 10%L_o_ was applied at a constant velocity of 1 L_o_•SL•s^−1^. (ii) For the experiments with blebbistatin, the fibers were divided in two sub-groups, treated with either an active form of blebbistatin (n = 12) or an inactive (control) form of blebbistatin (+/+) (n = 4). The fibers were first activated at a pCa^2+^ of 4.5 and shortened by 10%L_o_, at a velocity of 1 L_o_•SL•s^−1^; this trial provided the control value for these experiments. Following, the fibers were incubated in relaxing solution (pCa^2+^ = 9.0) containing blebbistatin. After the incubation period in relaxing solution, the fibers were immersed in activating solution (pCa^2+^ = 4.5) containing blebbistatin (5 µM). After full force development, a shortening of 10%L_o_ at 1 L_o_•s^−1^ was applied to the fibers. (iii) For the experiments with different velocities of shortening (n = 12), fibers were activated at pCa^2+^ of 4.5 or 6.0 (random order) and then immersed into relaxing (pCa^2+^ 9.0) and activating (pCa^2+^ 4.5) solutions containing blebbistatin (5 µM), as previously described. When force was fully developed, ramp shortenings of 10%L_o_ were applied at velocities of 0.125, 0.25, 0.5, 1.0, or 2.0 L_o_•s^−1^ (random order).

In all experiments, control contractions (pCa^2+^ of 4.5) were elicited throughout the experiments; when isometric forces decreased by >10% from the maximal force produced at the beginning of the experiment or when the striation pattern corresponding to the SL was lost during activation, the experiment was ended and data from this fiber was discarded from future analysis.

### Data analysis

The changes in slope of force observed during shortening were detected using a two-segment piecewise regression, in which the force trace can be fitted by two linear regression functions: *y_1_ = a_1_+b_1_x_i_* (restriction: *x_i_≤x_0_*), and *y_2_ = a_2_+b_2_x_i_* (restriction: *x_i_>x_0_*), where *(x_0_,y_0_)* represents the coordinates of the critical transition (L_c_ and P_c_ measured in this study), *a_1_* and *a_2_* represent the intercepts of the two regression lines, and *b_1_* and *b_2_* are the slopes of the two regression lines. At the first iteration, the observations *x_1_, x_2_, … x_5_* are included to estimate the parameters of the first regression line. The remaining observations *x_6_, … x_n_* are used to fit the second regression line. At the next iteration, the observations *x_1_, … x_6_* are included to estimate the parameters of the first regression line, and the remaining observations *x_7_, … x_n_* are used to fit the second regression line. The same procedure is performed in all iterations. The residual sum of squares (RSS) is based on the sum of the squares of each regression line:
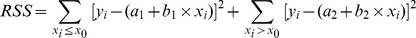
The RSS is used to determine the optimal values of *a_1_*, *a_2_*, *b_1_*, *b_2_*, and *x_0_* – the values associated with the minimal RSS are considered optimal [21]. The regression results were accepted when they presented a correlation coefficient (r*^2^*)>0.99, and data points fitted inside a 95% confidence interval (CI) of the regression lines, similar to procedures used previously in our laboratory [16]. In cases in which these criteria were not fulfilled, we used two approaches to detect the breakpoints, two single regression lines were fitted into the two slopes, which were extrapolated visually to detect the breakpoint [5], [16], [22]. Regression lines were accepted when the correlation coefficient (r^2^) were >0.99 and data points fitted inside a 95% confidence interval. The force produced during the first and second changes in slope were calculated at the transition points (P_1_ and P_2_, respectively). L_1_ and L_2_ were determined as the length change amplitudes necessary to achieve the P_1_ and P_2_ transitions, measured from the beginning of shortening. In a few experiments, the signal from the striation pattern arising from the fibers became weak during shortening; in this case, the L_1_ and L_2_ were calculated by extrapolating the percentage of change in fiber length (measured during the experiments) based on the SL measured just before shortening. Both methods provided similar results, as confirmed in experiments in which SL was measured.

The values of P_1_, P_2_, L_1_, and L_2_ were compared among different pCa^2+^ or blebbistatin conditions using a one-way ANOVA for repeated measures. When significant changes were observed, post-hoc analyses were performed with Newman-Keuls tests. For the third group of experiments a two-way ANOVA (3 conditions×5 velocities) for repeated measures were used to compare the P_1_, P_2_, L_1_ and L_2_. When significant interactions or main effects were found, post-hoc comparisons using Newman-Keuls adjustment for multiple comparisons were performed to locate significant differences. The significance level for all statistical tests was set at *P*<0.05.

### Model development

We developed a cross-bridge model consisting of two major elements: (1) an active molecular contractile element, consisting of cross-bridges capable of contraction and (2) a passive element with linear elasticity, which is placed between the active contractile element and the external apparatus which controls fiber length.

### Cross-bridge kinetics

**Figure 1:**
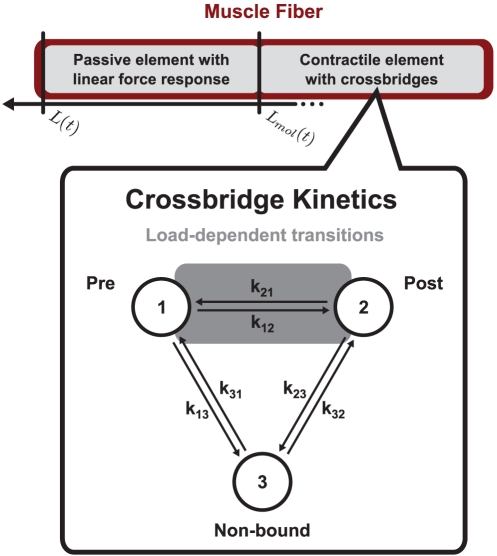
Overview of mathematical model. The mathematical model comprises a load-sensitive active cross-bridge component adjusting the molecular contractile apparatus length L_mol_, and a passive element with a linear force response to differences between the externally set fiber length L and L_mol_. A three-state cross-bridge kinetic cycle with a load-dependent power-stroke transition from the pre to the post-power-stroke state is assumed.

We assumed that the number of cross-bridges in the fiber is high enough to support a treatment of cross-bridge populations, instead of monitoring single cross-bridges. According to a simple Attach — Power-stroke — Detach scheme (Figures 1 and S1), we monitored three general populations of cross-bridges: *x_1_* – pre-power-stroke, *x_2_* – post –power-stroke, *x_3_* – non-bound (Figure 1). Cross-bridge transition between these populations occur at rates *k_ij_*; *i = 1,2,3* is the source population where the cross-bridge comes from, and *j = 1,2,3* is the sink population which the cross-bridge transits into. Based on these transition rates, we can describe the cross-bridge population dynamics in a set of two ordinary differential equations (ODEs):
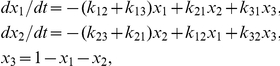
where *x_i_* represent the fraction of the total cross-bridges that can be found in the kinetic state *x_i_*. We used the normalization *x_1_+x_2_+x_3_ = 1* to reduce the system from three ODEs to two ODEs, because *x_3_* can be calculated from *x_1_* and *x_2_* as described in above expressions. All transition rates except those containing the power-stroke transition were assumed to be constant. The transitions containing the power-stroke have an exponential dependence on load *F*, which is multiplied by the power-stroke step size Δd and enters as work into the exponent:
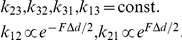
For exact expressions in terms of ATP, ADP, P_i_ concentrations, myosin affinity for actin, and zeroth order transition rates, see Text S1. Ionic strength, [Ca^2+^] and pH are assumed to be implicitly contained in the effective zeroth order rates for all transitions.

### Measured force

**Figure 2:**
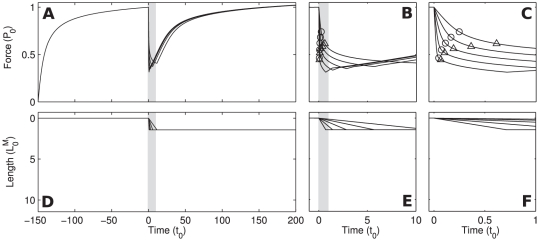
Simulated force during ramp shortening protocol with detected critical points. Top row (A, B, C) shows measured force P vs. time, bottom row (D, E, F) shows fiber length L vs. time. Triangles and circles represent P_1_ and P_2_ critical points, respectively. Negative times correspond to times before start of shortening ramp, fiber activated at time −150t_0_ in simulation and held isometrically up to time 0. Grey background rectangles indicate regions which are displayed at higher time resolution in the next graph to the right. Five traces are simulated with ramp velocities 0.125L_0_/t_0_, 0.25L_0_/t_0_, 0.5L_0_/t_0_, 1L_0_/t_0_ and 2L_0_/t_0_. Simulation parameters are presented in Text S1.

The measured force P is determined by the stretch of the passive elastic element. This stretch, in turn, is determined by the difference between *L*, the overall length of the fiber, and *L_mol_*, the length of the molecular contractile apparatus multiplied by the elastic modulus 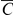:
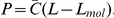
The fiber length L is externally set by the operator; we use the following time course to model a shortening ramp
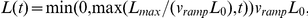
so that the ramp starts at time *t = 0*, the length *L(t = 0) = 0* is set equal *0* for the maximum isometric contraction point at *t = 0* (see Figures 2 D, 2E, and 2F). The fiber length *L* is changed at a constant velocity *v_ramp_* up to a total shortening length by *L_max_*. Note that in case of shortening *v_ramp_*, *L_max_<0*.

### Connecting cross-bridges and measured force

The active contractile element and the passive elastic element interact in two ways:

1. The force *P* on the fiber, which develops in the passive elastic element in response to stretch, is divided by the number of attached myosin heads, and thereby gives the load *F* experienced by a single attached myosin head
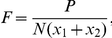
which in turn influences the transition rates of the forward and reverse power-stroke steps. *N* is the total number of cross-bridges in the fiber.
2. Cross-bridges transitioning through the forward or reverse power-stroke step decrease or increase *L_mol_* (the length of the active molecular contractile element), respectively. The change in *L_mol_* can be calculated by multiplication of the forward flux through the power-stroke state by the power-stroke step size
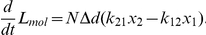

*L_mol_*, together with *L*, determines the stretch of the passive elastic element, and thereby the measured fiber force.

### Implementation of experimental protocol, measurement of critical points

To reproduce the experimental ramp shortening protocol in a simulation of our model, we allowed the fiber to reach a force plateau (P_0_) before imposing a ramp shortening (Figure 2A). Dependent on ramp velocity, the time of the ramp shortening was adjusted in each contraction to reach the same ramp lengths independent of ramp velocity (see Figures 2D and 2E). We simulated our model using the MatLab ode15s adaptive time step size integrator for stiff ODEs (for resulting traces see Figures 2 and S2). We found regular ODE integrators to be inefficient due to the rapid changes in force right after beginning of the ramp shortening. The P_1_ and P_2_ transitions were detected based on the curvature *Curv* of the force time course *L(t)*. At P_1_, the linear force decrease transitions into a less steep decrease, *Curv* at this “kink” has a prominent peak which we used to detect P_1_; considering P_1_ as a transition from a phase with marked shifts in the cross-bridge populations to a phase of exponential approach to a new steady state, we detected P_2_ as a characteristic transition in *log10(Curv)* from a curved decay to a linear decay (exponential decay displays as a linear decay on a log-scale) (see Figures 2 and S3).

## Results

### Experimental results

The isometric forces were altered by Ca^2+^ concentration changes as well as by blebbistatin (Table 1). When contractions were produced in pCa^2+^ 5.0 or 5.5, a not statistically significant trend towards decreased force was visible, and contractions in pCa^2+^ 6.0 showed a significant decrease in force relative to contractions produced at pCa^2+^ 4.5. When fibers were treated with blebbistatin, there was a significant force decrease, in accordance with previous studies that reported a decrease of ∼60% when using 5 µM of blebbistatin [7], [16], [23]. The effects of blebbistatin are highly dependent on the experimental conditions; differences of ∼20% are observed in studies that use similar blebbistatin concentrations [7], [23]. Note that the inactive isomer of blebbistatin (+/+) also decreased the force by a small magnitude, a result that has been reported previously [16], [23]. However, during shortening, we did not find any difference between the inactive form of blebbistatin and the control experiments, as previously observed [16]. For reasons of clarity we will therefore report only the results of experiments using the active form of blebbistatin.

**Table 1:** Relative force decrease (%) in contractions produced in the different experimental conditions, when compared to contractions produced at pCa^2+^ 4.5.

	Experiment		
	(i)	(ii)	(iii)
pCa^2+^ 5.0	20±15	-	-
pCa^2+^ 5.5	18±17	-	-
pCa^2+^ 6.0	59±5*	-	55±10*
Blebbistatin	-	60±6*	72±4*

Legend: (i) first set of experiment, with fibers activated at four Ca^2+^ concentrations, (ii) second set of experiments, with fibers activated in presence or absence of blebbistatin, and (iii) third set of experiments, with fibers activated in pCa^2+^ 4.5 (with and without blebbistatin) and pCa^2+^ 6.0. The three sets of experiments correspond to the description given in Methods.

*Significantly different from contractions produced at pCa^2+^ 4.5 (p≤0.05).

#### Effects of Ca^2+^ concentrations

**Figure 3:**
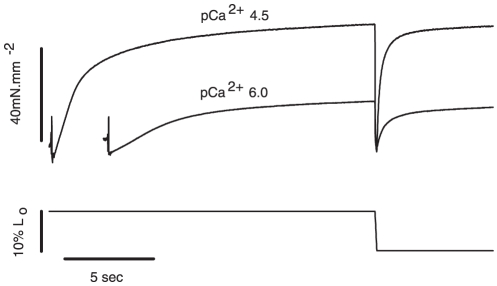
Typical experiment overview – pCa^2+^4.5 and 6.0. Sample records from a typical experiment showing the force produced by a muscle fiber activated in pCa^2+^ 4.5 (upper trace) and pCa^2+^ 6.0 (lower trace). Force rises during activation and then stabilizes to achieve a plateau. During shortening the force decays rapidly. After the shortening, the forces recover slowly to achieve a new steady-state.

Figure 3 shows two contractions (pCa^2+^ 4.5 and 6.0) recorded during a typical experiment performed in this study. In both cases the force rose quickly during activation to reach different steady-state levels – in this case the force produced at pCa^2+^ 6.0 was 40% of the force produced at pCa^2+^ 4.5. Once full force development was obtained, the fiber was shortened and the force rapidly decreased to zero. The force was then redeveloped to reach a new steady state, after which the fiber was deactivated (deactivation not shown).

**Figure 4:**
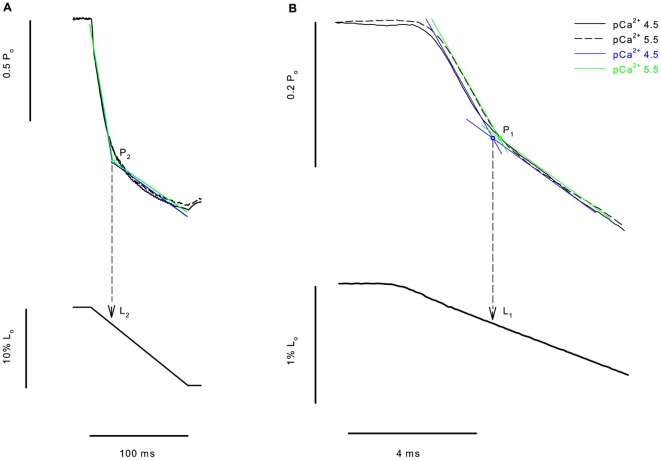
Experimental detection of critical points at different Ca^2+^ concentrations. (A) Superimposed contractions showing the force decrease during shortening while the fiber was activated at different Ca^2+^ concentrations (top), with the corresponding length change (bottom). All forces were normalized by their respective isometric forces (P_o_) before the ramp shortening. P_2_ and L_2_ did not change at increasing Ca^2+^ concentrations. (B) Closer view from the initial shortening phase of the experiment with another fiber, showing clearly that P_1_ and L_1_ do not change with different Ca^2+^ concentrations. The critical points in this figure were detected with regression analyses; the regression lines are shown in blue (pCa^2+^ 4.5) and green (pCa^2+^ 5.5) traces.

**Figure 5:**
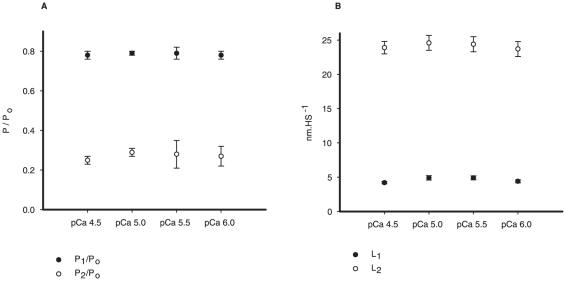
Mean critical values for different Ca^2+^ concentrations. Mean values (± S.E.M) of P_1_ and P_2_ (A), and L_1_ and L_2_ (B) in experiments performed with different Ca^2+^ concentrations. Changing the pCa^2+^ did not change any of the variables.

We were mostly interested in the transient force changes during shortening. Figure 4A shows a zoomed image of the shortening phase during two contractions produced in different pCa^2+^. The force was normalized by the maximum isometric force produced just before shortening. In this case the values of P_2_ and L_2_ were not different among the different contractions. In Figure 4B we changed the graph scale to show the P_1_ force transition, which was clearly detected. The values of P_1_ and L_1_ did not change during the shortening with different pCa^2+^. The results observed in this experiment were confirmed statistically, and despite the increase in P_o_ following the increase in Ca^2+^ concentrations, none of the variables investigated during shortening (P_1_, P_2_, L_1_ and L_2_) were affected by changes in pCa^2+^ (Figure 5). When all pCa^2+^ data were pooled, P_1_ and P_2_ were 0.79±0.003 and 0.27±0.01 times P_o_, respectively, and L_1_ and L_2_ were 4.62±0.16 and 24.17±0.20 nm•HS^−1^ respectively.

#### Effects of blebbistatin

**Figure 6:**
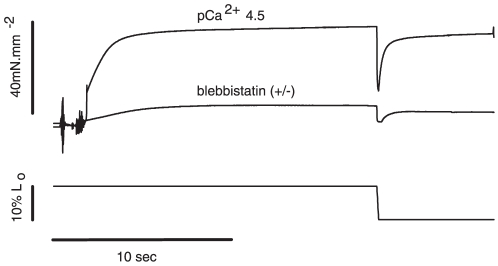
Typical experiment overview – pCa^2+^4.5 and blebbistatin. Sample records from a typical experiment showing the force produced by a muscle fiber activated in pCa^2+^ 4.5 (upper trace) and then treated with blebbistatin (lower trace). Force rises during activation and then stabilizes at a steady-state level. During shortening the force decreases. After the shortening, the forces recover slowly to achieve a new steady-state.

**Figure 7:**
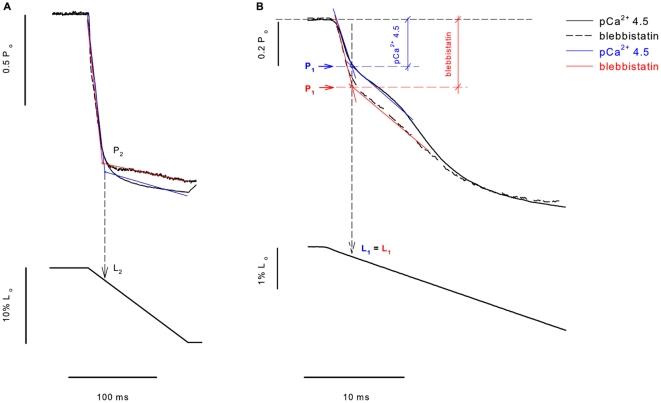
Experimental detection of critical points in fibers treated with blebbistatin. (A) Superimposed contractions, showing the force decay during shortening while the fiber was activated with pCa^2+^ 4.5- (solid line), and then treated with blebbistatin (dashed line), with the corresponding fiber length changes. All forces were normalized by their respective isometric forces (P_o_) before shortening. P_2_ and L_2_ did not change with blebbistatin. (B) Closer view from the initial shortening phase of the experiment with another fiber, showing that blebbistatin induced a higher force decrease before P_1_. It also shows that blebbistatin induced greater P_1_ amplitude when compared the contraction produced before blebbistatin. L_1_ was not changed by blebbistatin. The critical points in this figure were detected with regression analyses, the regression lines are shown in blue (pCa^2+^ 4.5) and red (pCa^2+^ 4.5+blebbistatin) traces.

**Figure 8:**
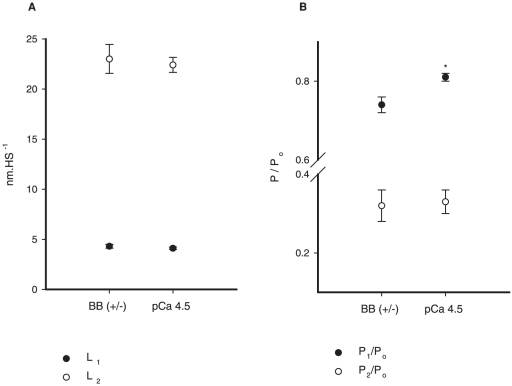
Mean critical values of fibers treated with blebbistatin. Mean values (± S.E.M.) of P_1_ and P_2_ (A), and L_1_ and L_2_ (B) in experiments where fibers were treated with blebbistatin. Blebbistatin changed P_1_ significantly. None of the other variables were changed. * Significantly different from all other conditions (P<0.05).

Figure 6 shows two contractions recorded during the same experiment before and after blebbistatin treatment. Blebbistatin substantially decreased the maximum isometric force, but the response to shortening was similar to control experiments: the force decreased quickly to almost zero to then redevelop towards a new steady-state level. Figure 7 shows a zoomed image of the shortening phase in an experiment where the fiber was activated at pCa^2+^ 4.5 and treated with blebbistatin (+/−). The force was normalized by the maximum isometric force. The values of P_2_ and L_2_ were not different before and after blebbistatin treatment (Figure 7A). However, P_1_, also detectable for this condition, decreased significantly after blebbistatin treatment, while L_1_ was not changed (Figure 7B). The results shown in the experiment depicted in Figure 7 were confirmed statistically (Figure 8). P_2_ and L_2_ were not statistically different from the control group (pCa^2+^4.5). The P_1_ amplitude (absolute distance between the critical point and P_o_) was significantly higher after blebbistatin treatment when compared with the control, as shown in a higher force decrease, while L_1_ was not affected by blebbistatin (Figure 8B).

#### Effects of shortening velocity

**Figure 9:**
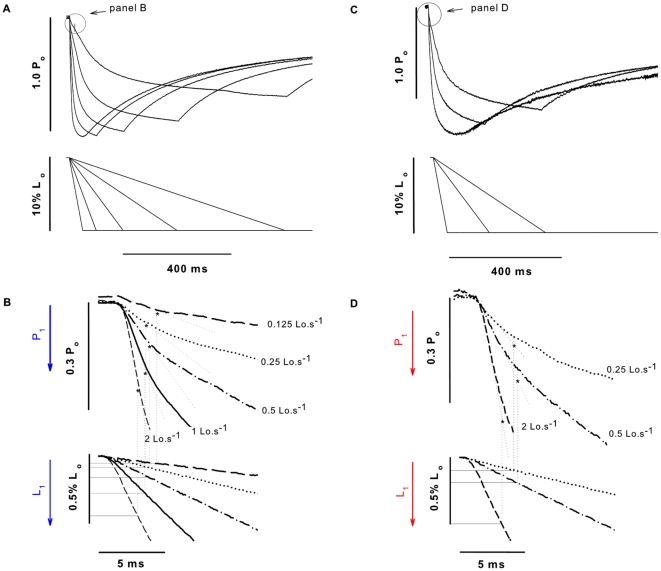
Critical points behaviour at different velocities. (A) Force responses (upper traces) to ramp shortenings (lower traces) in a range of velocities (0.125–2 L_o_.s^−1^) from one set of experiments performed with one fiber activated with a pCa^2+^ 4.5. The rate of force decay increased with shortening velocities. (B) Closer view from the same traces, showing increasing P_1_ amplitude (upper traces) and L_1_ (lower traces) with increasing velocities. (C) and (D) Same as in *A* and *B*, now showing traces of another fiber treated with blebbistatin (3 velocities displayed).

**Figure 10:**
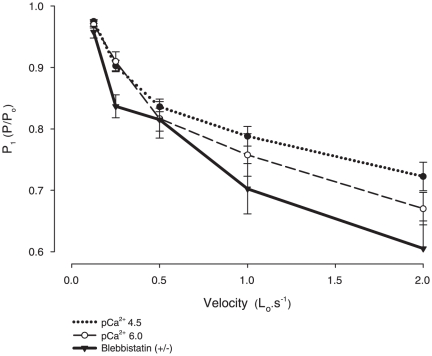
Mean critical values at different velocities. Mean (± S.E.M.) of the P_1_ values at five different velocities in a fiber activated with pCa^2+^ 4.5 (dotted line), 6.0 (solid line) and treaded with blebbistatin (solid line with inverted triangles). Blebbistatin changed P_1_ significantly at all five velocities. *Significantly different from all other conditions (P<0.05).

Figures 9A and 9C show records of contractions in which ramp shortenings at different (constant) velocities were applied in two fibers activated in pCa^2+^ 4.5 and treated with blebbistatin, respectively. The P_2_ amplitude and L_2_ were augmented with increasing velocities, as previously shown [5]. Figures 9B and 9D show a closer view of the force records from panels A and C respectively in which the P_1_ transitions were detected. The amplitude of P_1_ increased with velocity, which was accompanied by an increase in L_1_. The amplitudes of P_1_, P_2_, L_1_, and L_2_ increased with velocities, in all conditions investigated. There was not a difference detected in P_2_, L_1_, and L_2_ among the three conditions, although the P_1_ amplitude increased in fibers treated with blebbistatin. Figure 10 shows the mean (± S.E.M) values for P_1_ activated in pCa^2+^ 4.5, 6.0 and after treatment with blebbistatin. The velocity-P_1_ curve was shifted downward in fibers treated with blebbistatin.

### Model results

**Figure 11:**
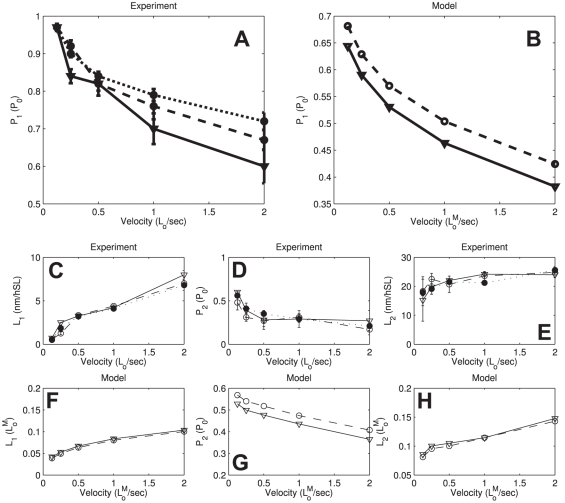
Blebbistatin effect on ramp shortening critical points in experiment and model simulation. (A) Experimentally measured P_1_ for different ramp velocities. Solid line: pCa^2+^ 4.5 with blebbistatin, dotted and dashed line: pCa^2+^ 4.5 and pCa^2+^ 6 without blebbistatin, respectively. (B) P_1_ detected in simulation for different ramp velocities. Solid line: blebbistatin inhibition modeled by lowering of myosin actin tight binding energy by ΔE = 0.35k_B_T. Dashed line: no blebbistatin inhibition. (C, D, E) Experimentally determined L_1_, P_2_, L_2_, respectively; same conditions as in (A). (F, G, H) L_1_, P_2_, L_2_ detected in simulated ramp shortening, respectively; same conditions as in (B). For simulation parameters see Text S1.

Using our model (for parameter values see Table 1 in Text S1), the dependence of P_1_, P_2_, L_1_, and L_2_ on ramp velocity observed in our experiments without blebbistatin could be qualitatively explained (Figure 11); the critical P_1_ and P_2_ decreased and the critical L_1_ and L_2_ increased with increasing ramp velocity. We also observed the characteristic nonlinear dependencies of the critical force transitions during the shortening; the monotonous upward and downward tendencies observed in experiment for P_1_, P_2_, L_1_, and L_2_ are all accounted for by our model results.

#### Mechanism of blebbistatin inhibition

**Figure 12:**
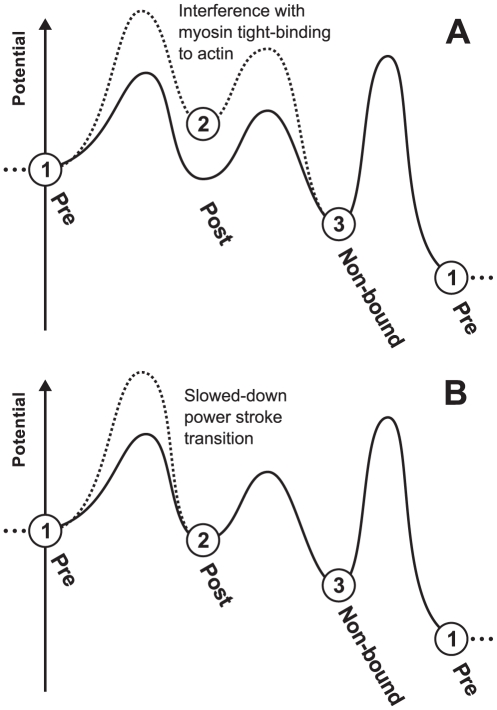
Molecular mechanism of blebbistatin inhibition visualized in the cross-bridge cycle potential profile. We display here the two suggested mechanisms of blebbistatin inhibition, each with its specific effect on the potential profile. The solid curve represents the free energy profile without blebbistatin inhibition; the dashed curve represents the qualitative change from blebbistatin addition. A progression through states 1, 2, 3 and finally back to 1 (from left to right) corresponds to completion of one actomyosin cross-bridge cycle by sequential transition through the kinetic states. The elevations between the kinetic states correspond to reaction barriers; these transitions require activation energy, so a higher barrier lowers the transition rates across this barrier. (A) Reduction of binding energy of myosin tight-binding to actin, manifesting itself as an increase of the post-power-stroke energy level. Our model analysis indicates that this is a necessary mechanism of blebbistatin inhibition. (B) Reduction of the power-stroke zeroth order rate constant. Our model analysis indicates that this is a possible but not a necessary mechanism of blebbistatin inhibition. Potential profiles are only qualitative illustrations and not drawn to scale.

The power-stroke inhibitor blebbistatin is believed to affect the tight binding of myosin to actin. Its characteristic molecular structure targets the myosin-actin binding interface, and the closing of the myosin's actin-binding cleft is hindered. Conceptually, there are two not mutually exclusive ways to incorporate this mechanism into cross-bridge kinetics: (1) as the establishment of inter-protein molecular bonds is disturbed by the presence of blebbistatin, the binding energy of the tight-bound post-power-stroke state is reduced, or (2) as the closing of the actin binding cleft is hindered, the zeroth order transition rate of the power-stroke is reduced. In terms of the molecular potential energy profile, these changes correspond to an increase of the potential energy level of the pre-power-stroke state, or the increase of the reaction energy barrier of the transition between the pre and the post-power-stroke state, respectively (see Figure 12). Intuitively, case (1) would be expected to lower the effective affinity between actin's myosin binding sites and myosin heads. Case (2) would be expected to slow down the power-stroke transition, but not to change the effective myosin-actin affinity. Both alterations are in line with findings from biochemical and structural studies of kinetic mechanism of blebbistatin [8], [24]–[26]. However, we can show that in case (2) no reduction in the isometric maximum force P_o_ is predicted with blebbistatin. In case (1), P_o_ decreases monotonously with increasing concentration of blebbistatin. Thus, a decreased binding energy of the strongly bound cross-bridges as described under (1) is the necessary mechanism of action and used for the following model predictions of the blebbistatin effect.

#### Prediction of critical points with blebbistatin inhibition

We introduced the kinetic influence of blebbistatin into our model as a reduction of the binding energy by ΔE = 0.35k_B_T. This alteration predicts a prominent reduction of the P_1_ and P_2_ and minimal changes in L_1_ and L_2_. Applying the power-stroke inhibitor blebbistatin in our experiment caused a significant decrease in P_1_ force and no significant changes for P_2_, L_1_, and L_2_. Thus the predicted and measured qualitative effects of blebbistatin are the same for P_1_, L_1_ and L_2_; the case of P_2_ is unclear. Applying alternatively an increase in the zeroth order rate constant of the power-stroke by a factor 1/1.75 predicts similar effects on the critical points (Figure S4), thus a decrease in the energy of tight binding of myosin to actin is a necessary mechanism to explain our results, the reduction of the zeroth order power-stroke transition rate is a possible one.

## Discussion

In this study we detected an early force transition during an imposed shortening of activated muscle fibers (P_1_), depicted as a change in slope prior to P_2_ during shortening [1], [2], [5]. In our experiments, when different velocities (0.125 to 2 L_o_•SL•s^−1^) were applied, P_1_ amplitude (i.e. distance between Po and critical force) ranged between −0.03 and −0.39 times P_o_ and L_1_ amplitudes ranged between 0.5 and 7.0 nm•HS^−1^; the higher the velocity the higher P_1_ and L_1_ amplitude; in agreement with previous studies [5]. We also observed values for P_2_ and L_2_ that were within the range observed in previous studies; when a 10%L_o_ shortening was performed at velocities ranging from 0.125 to 2 L_o_•SL•s^−1^, the values of P_2_ and L_2_ ranged between ∼0.65 and 0.20 times P_o_ and ∼15 and 27 nm•HS^−1^.

### Experiments with different Ca^2+^ concentrations

Despite the velocity dependence of P_2_, the values did not change with Ca^2+^ concentrations (pCa^2+^ 4.5, 5.0, 5.5 and 6.0), suggesting that P_2_ values are independent from the number of cross-bridges attached to actin. Although estimating the number of strongly-bound cross-bridges in a given moment during contractions is challenging, there is evidence that the number does not exceed 40% at high Ca^2+^ concentrations. One study that experimented with permeabilized fibers from the rabbit psoas muscle, and used stiffness measurements, to calculate the relative proportion of cross-bridges attached to actin, found a value of ∼33% of cross-bridges attached to actin during isometric contractions produced at saturating Ca^2+^ concentration [27]. It is likely that the number is similar to what we have in our experiments.

To our knowledge, no other studies evaluated the effects of Ca^2+^ concentrations on P_2_ during shortening. If we assume that the amount of cross-bridges formed before shortening do not change the strain necessary for their detachment, and the critical forces (P_2_) normalized by their isometric forces, as well as their correspondent critical length (L_2_), should not change. Previous studies that evaluated P_1_ during shortening [5] have suggested that this early inflexion is born mostly by pre-power-stroke cross-bridges (newly attached cross-bridges) performing the power-stroke. Assuming that increasing Ca^2+^ concentration alters forces mostly by increasing the number of cross-bridge formation, without necessarily affecting the distribution of the population of myosin attached to actin into pre and post-stroke states, it is expected that P_1_ would not change with different Ca^2+^ concentrations.

### Modelling cross-bridge kinetics

The developed model explains qualitatively L_1_, P_1_, L_2_, and P_2_ for different ramp velocities. A parameter change representing the known kinetic effects of blebbistatin also predicts the experimentally observed L_1_, P_1_, and L_2_. Furthermore, on the level of actomyosin interaction, the model is mechanistic and based on experimental results. Together, these findings imply that important aspects of the underlying molecular kinetics are captured in the model. It therefore seems appropriate to extend our investigation of the cross-bridge kinetics underlying the ramp shortening force response using this model.

**Figure 13:**
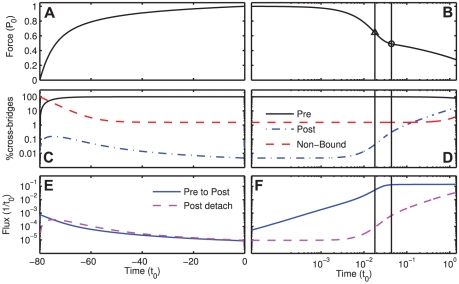
Simulated cross-bridge dynamics during ramp shortening protocol with ramp velocity 2L_0_/t_0_. A logarithmic time scale was used for all positive times; time 0 indicates start of ramp shortening, negative times indicate isometric contraction phase before ramp shortening. (A, B) Force production, triangle and circle represent critical points P_1_ and P_2_, respectively. (C, D) Percentage of actively cycling cross-bridges in the different kinetic states. Note logarithmic scaling of vertical axis. (E, F) Effective flux of cross-bridges from pre- to post-power-stroke-state and from post-power-stroke state to detached state. The effective flux is the rate at which cross-bridges go from a state x_i_ to a state x_j_ minus the rate at which cross-bridges go from x_j_ to x_i_. Note logarithmic scaling of vertical axis. See also Discussion section and Text S1; simulation parameters are included in Text S1.

It has been hypothesized in earlier experimental and model studies that the P_1_ critical point is associated with a transition from a purely elastic phase to a repartitioning of the cross-bridge populations in response to a decreased load on the bound cross-bridges. As can be seen in Figure 13, short after the ramp shortening started, only a minimal change in the cross-bridge populations was visible. This corresponds to a phase in which only the passive elastic elements in the muscle fiber are shortened, which decreases the measured force as well as the load on the bound cross-bridges. When P_1_ is reached, the load on bound cross-bridges is decreased so far as to appreciably increase the power-stroke forward transition rate. As an effect, the pre-power-stroke cross-bridges go through the power-stroke fast, and an increasing percentage of cross-bridges appears in the post-power-stroke and the non-bound state. This shift in the cross-bridge populations first increases, and then decreases again till the cross-bridge populations reach a new steady state for the changed loading conditions. The behaviour of the cross-bridge populations visible in Figure 13 around the second critical point at P_2_ suggests an association of P_2_ with the transition from an increasing to a decreasing shifting behaviour in the cross-bridge populations, resulting in an asymptotic approach to a new, lowered steady state tension value.

### Mechanism of blebbistatin inhibition

When the fibers were treated with blebbistatin, a decrease in the maximum isometric force P_0_ was observed. There was a significant decrease in P_1_, but no significant changes in L_1_, P_2_ and L_2_ after blebbistatin treatment. Blebbistatin is a myosin inhibitor that causes both, a reduction in the number of cross-bridges strongly attached to actin, and a redistribution of cross-bridges towards a weakly bound state, stabilizing the myosin•ADP•P_i_ complex into a pre-power-stroke state [7], [8], [24]. Investigation of our model indicated that a decrease in the binding energy of the myosin-actin tight binding is a necessary molecular mechanism of blebbistatin inhibition of active force development, in contrast to a reduction in the zeroth order rate constant of the power-stroke transition it can explain the P_0_ reduction. This is in agreement with the hypothesized disturbance of the tight binding protein-protein interface [7], [8], [24]. Decrease of the strength of tight binding as well as a reduction of the zeroth order rate constant of the power-stroke transition were found to explain the observations for P_1_, L_1_, and L_2_.

Since we observed that blebbistatin affects some of the contractile parameters during shortening and varying Ca^2+^ concentration had no effect, it is important to discuss the difference between force inhibition by blebbistatin and by the Ca^2+^-troponin-tropomyosin complex. According to the most accepted model of force regulation [28], when Ca^2+^ binds to the troponin C (TnC), it causes the displacement of tropomyosin, allowing cross-bridge attachment to actin and forming a weakly bound myosin–actin–ATP complex. ATP is then hydrolysed and phosphate is released, forming a strongly bound myosin–actin–ADP complex. The strongly bound complex causes conformational changes in the thin filament, increasing the probability of new cross-bridges to attach to actin. Therefore, the troponin-tropomyosin complex regulates force production mostly by not allowing myosin-cross bridges to attach to actin, while blebbistatin decreases the formation of the strong-bound step, after the formation of the myosin•ADP•P_i_ complex.

### Indications for a load-dependent ADP-release step

Our model predicts surprisingly low occupancies of the post-power-stroke state at maximal isometric contraction (see Figure 4 and a more general derivation in Text S1). While maintenance of maximal isometric force by mostly pre-power-stroke cross-bridges is not necessarily wrong, the large presence of pre-power-stroke cross-bridges disagrees with X-ray diffraction studies showing ∼40% of cross-bridges in the (stereospecifically bound) post-power-stroke state during isometric contraction at physiological temperatures [29]. However, when we include the stress-sensitivity of ADP release hypothesized for skeletal muscle [30], a non-vanishing percentage of cross-bridges in the post-power-stroke state for maximal isometric force becomes possible (see Text S1). Therefore, the strain-sensitivity of the ADP release might play a greater role in ramp force responses than appreciated so far. From the first comprehensive models of muscle molecular mechanochemistry [12] to more recent modeling studies [31], a strain-sensitive ADP release has been assumed, thus it should be an interesting future direction for more realistic models of skeletal muscle cross-bridge kinetics in ramp shortening and lengthening experiments.

## Supporting Information

Figure S1 **Cross-bridge kinetic scheme used in the general model.** Three kinetic states are assumed: (1) a pre-power-stroke state, (2) a post-power-stroke state, and (3) a non-bound state, in which myosin is not attached to actin. The transition ratefunctions k_ij_ displayed with the arrows between the kinetic states characterize the dynamic behavior of the cross-bridge population. Solid arrows indicate transitions, which are part of the regular cross-bridge cycle. The dashed line is a transition stemming from reverse stretch of pre-power-stroke myosin, which detaches from actin without completing the regular cross-bridge cycle. The transition associated with rate k_13_ contains contributions from the regular cross-bridge cycle as well as “ripping”, i.e. forced detachment of myosin cross-bridges from stretch on the fiber. In the main paper, we use a reduction of this model, where all parameters related to this forced cross-bridge “ripping” are set equal 0.
(EPS)

Figure S2 **Ramp shortening and lengthening model traces.** The characteristic P1 and P2 transitions for ramp shortening (traces with force decrease during ramp) and lengthening (traces with force increase during ramp) become visible from simulation of the general model. For features observed in experiment see Pinniger et al. and Roots et al. [5], [7] in Text S1. (A) Force vs. time for the complete experimental protocol including isometric contraction at negative times and a ramp stretch beginning with time *t = 0* and ending when maximal ramp length is reached. (B) Zoom into (A) to make visible features of the overall force response including the P2 features of lengthening and shortening and the P1 characteristic indentation of the lengthening force response. (C) Zoom of A) to make visible the early force response including the P1 characteristic force drop right after beginning of a ramp shortening. Ramp velocities *V_ramp_ = −2L_0_^M^, −1L_0_^M^, 1L_0_^M^, 2L_0_^M^*; simulation parameters given in table in Text S1, general model.
(EPS)

Figure S3 **Detection of critical points from simulated force responses to ramp shortening.** In all graphs heavy lines represent a simulation for *V_ramp_ = 2.0L_0_*, the regular lines are for *V_ramp_ = 1, 0.5, 0.25, 0.125*, in respective order away from the heavy line. (A) *L(t)* (solid black) and *L_mol_(t)* (dashed red) in units of *L_0_* vs. time in units of *t_0_*. After starting the ramp *L* decreases, and in effect *L_mol_* becomes more similar to *L*, which effects a force decrease. (B) Force response to ramp shortening. Circles indicate the *P_1_* transition, triangles the *P_2_* transition. (C) Percentage of cross-bridges in pre-power-stroke (solid black), post-power-stroke (dotted blue) and Non-bound (dashed red) State. (D) *log_10_* of the curvature *Curv* of the force response. Circles indicate the detection points of *P_1_* at the first curvature maximum. (E) First derivative with respect to time of *log_10_(Curv)*. (F) Second derivative with respect to time of *log_10_(Curv)*. Triangles indicate the detection points of *P_2_* at the first maximum with a negative value in the first time derivative.
(EPS)

Figure S4 **Blebbistatin effect on ramp shortening critical points in experiment and model simulation assuming reduction of the power-stroke zeroth order rate constant.** (A) Experimentally measured P1 for different ramp velocities. Solid line: pCa4.5 with blebbistatin, dotted and dashed line: pCa4.5 and pCa6 without blebbistatin, respectively. (B) P1 detected in simulation for different ramp velocities. Solid line: blebbistation inhibition modeled by reduction of the zeroth order power-stroke-rate constant *k_0_' = k_0_/1.75*. Dashed line: no blebbistation inhibition. (C, D, E) Experimentally determined L1, P2, L2, respectively; same conditions as in (A). (F, G, H) L1, P2, L2 detected in simulated ramp shortening, respectively; same conditions as in (B).
(EPS)

Text S1 **Cross-bridge model development and analysis.**
(PDF)
