# Supplementary material for: Pre-Power-Stroke Cross-Bridges Contribute to Force Transients during Imposed Shortening in Isolated Muscle Fibers
Source: PLoS One. 2012 Jan 5;7(1):e29356. doi: 10.1371/journal.pone.0029356 (PMC3252314; doi:10.1371/journal.pone.0029356)
Supplement: Text S1 — Cross-bridge model development and analysis. (PDF) [file pone.0029356.s005.pdf]

Cross-bridge model development and analysis

1 General approach and structure of appendix

This model serves as a simple approach to understanding the contributions of actin-myosin cross-bridges (simply called cross-bridges hereafter) in different kinetic states to the force transients observed in single fiber ramp stretch and shortening experiments. Other more comprehensive models for such studies exist, taking into account contributions of sarcomere non-uniformities and the behavior of other proteins than actin and myosin under the influence of ramp length alterations. Our focus, however, is only on the very early response to ramp stretch and shortening. The specific P1 and P2 features of force transients have been ascribed to mostly cross-bridge dynamics before[5, 7]. We can therefore explore in how far cross-bridge dynamics alone can explain the observed force transients.

To construct a simple, though realistic dynamical system to model the stretch and shortening response, we consider two features: (1) An active contractile element driven by the cross-bridge interaction between actin and myosin under dynamically changing loads, (2) A passive elastic element with linear force response to stretch, see Figure 1 in the main text.

First, we will introduce our general model. Second, we will evaluate our model numerically, including the examination of two possible ways to account for blebbistatin inhibition. This general model can be applied to simulate force response curves for ramp shortening and lengthening with different ramp velocities. Finally, using a simplified generic model, we will investigate the distribution into pre- and post-power-stroke cross-bridges at maximal isometric force and rule out one potential blebbistatin inhibition mechanism as a necessary one.

2 Model development

2.1 Active contractile element

Effectively cycling cross-bridges: Even at full activation of a muscle, not all cross-bridges are actively participating in the propulsion of thin filaments. At lower activation, even less cross-bridges take part in this action. We consider in our model only those cross-bridges which are effectively available for this interaction, often referred to as actively cycling cross-bridges. As an example, when inside our model we speak of almost 100% cross-bridges in states bound to thin filament, this really means that almost 100% of those cross-bridges that are actively cycling are also bound to a thin filament. This does not mean that all myosins present in thick filaments are bound to actin, as by our definition actually a great number of them could effectively not be cycling. In the following we will refer to the number of effectively cycling cross-bridges as N.

Kinetic states: We imagine a population of N effectively cycling cross-bridges, and assume that changes in their total number due to overlap and activation changes can

be neglected. These cross-bridges can be in three states: (1) weak-bound, pre-power-stroke state with ADP and P bound (2) tight-bound, post-power-stroke state with ADP bound, and (3) non-bound, i.e. without myosin attached but ATP or ADP.P bound to the myosin catalytic pocket. We assume a high number of cross-bridges, so instead of treating single cross-bridges, we follow the fraction of cross-bridges in each of the kinetic states. We name these fractions x_1, x_2 and x_3 for pre-power-stroke, post-power-stroke and non-bound, respectively. When these state occupancies are multiplied by the total cross-bridge number N, the number of cross-bridges in a specific state can be calculated. As any cross-bridge has to be in one of these three states, they sum up to 1:

$$x_1 + x_2 + x_3 = 1, \tag{1}$$

which allows us to express x_3 in terms of x_1 and x_2

$$x_3 = 1 - x_1 - x_2, \tag{2}$$

so we have effectively reduced our system to two kinetic states.

Transitions between states: Regular cross-bridge cycling can, in principle, proceed forward and backward, and the utilization of free energy from ATP hydrolysis biases this cycle into the forward direction[1]. As we assumed three kinetic states which are ordered into a single kinetic cycle, we have six transition rates between the kinetic states, see Figure S1. These define the dynamic behavior of the cross-bridge population with respect to their occupancy of kinetic states:

$$\dot{x_1} = -(k_{12} + k_{13})x_1 + k_{21}x_2 + k_{31}(1 - x_1 - x_2) \tag{3}$$

$$\dot{x_2} = -(k_{21} + k_{23})x_2 + k_{12}x_1 + k_{32}(1 - x_1 - x_2). \tag{4}$$

The transition rates are determined by their specific zeroth order rate constants, substrate concentrations and force applied to the cross-bridge:

$$k_{12} = k_P^0 \frac{1}{\sqrt{p}} \exp(-F\Delta d/2) \tag{5}$$

$$k_{21} = k_P^0 \sqrt{p} \exp(F\Delta d/2) \tag{6}$$

$$k_{23} = k_{det}^0 \sqrt{\frac{atp}{adp}} \frac{1}{K_{att}} \tag{7}$$

$$k_{32} = k_{det}^0 \sqrt{\frac{adp}{atp}} K_{att} \tag{8}$$

$$k_{31} = k_{att}^0 K_{att} \tag{9}$$

$$k_{13} = k_{att}^0 \frac{1}{K_{att}} + k_{rip}^0 \exp(F/\tilde{C}_{rip}). \tag{10}$$

In k_{12} and k_{21} the stress dependent term has been divided by 2 for an equal partitioning of stress dependence to the forward and backward power-stroke transition. This was chosen as the model results agree well with our data. In k_{13} a "ripping" term has been introduced which bypasses the regular kinetic cycle. When the muscle fiber is stretched, cross-bridges are stressed against their regular direction of cycling, and are therefore prone to forceful "ripping" off the actin filament without completion of the regular cross-bridge cycle. The cross-bridge "ripping" under stretch is expressed in the last term in k_{13}. These effective transition k^0 rates between the $\mathbf{x}$ states include the total number of cross-bridges:

$$k_{att}^0 = N\overline{k}_{att}^0,\ k_P^0 = N\overline{k}_P^0,\ k_{det}^0 = N\overline{k}_{det}^0,\ k_{rip}^0 = N\overline{k}_{rip}^0, \tag{11}$$

where the $\overline{k}_0$ are zeroth order transition rate constants for single cross-bridges.

Load dependence: The load dependence of life times (which are inverse proportional to transition rates) of kinetic states on external load has been shown for several slow myosin types[8, 4, 2]. The functional form of the stress dependence found therein corresponds with Hill's exponential load-dependence[1] for the power-stroke transition, which we employed in our model.

Normalized nucleotide and P concentrations: In the transition rate expressions we use normalized concentrations

$$atp = \frac{[ATP]}{[ATP_0]},\ adp = \frac{[ADP]}{[ADP_0]},\ p = \frac{[P_i]}{[P_{i,0}]}, \tag{12}$$

where no subscript denotes the actual concentrations in μM and the subscript 0 denotes the equilibrium concentration in μM. The equilibrium concentrations are related to the free energy of hydrolysis at standard conditions $[ATP] = [ADP] = [P_i] = 1$mM in the following way

$$\Delta G_0 = -\ln \frac{[ADP_0][P_{i,0}]}{[ATP_0]}, \tag{13}$$

where $\Delta G_0 < 0$ is the free energy of ATP hydrolysis of one ATP molecule in units of $k_B T$. According to [3] the free energy of ATP hydrolysis under physiological conditions is $\Delta G_0 = -12.34$ in units of $k_B T$ at laboratory standard temperature $T = 298.15\text{K} = 25°\text{C}$. The nucleotide concentrations atp and adp only occur as a ratio in our reaction rates, so we only need one equilibrium concentration to normalize them. Thus we set $[ATP_0] = 1$ without loss of generality. We get

$$\frac{atp}{adp} = \frac{[ATP][ADP_0]}{[ADP]}. \tag{14}$$

For equation (13) to be valid, the following relation must hold

$$[ATP_0] = [P_{i,0}]e^{-12.34}. \tag{15}$$

This shows that only the equilibrium concentration $[P_{i,0}]$ can be freely chosen in the framework of our model. Note that by this normalization all rate constants except those for forceful detachment are thermodynamically well-defined with respect to heat dissipation per cross-bridge cycle ΔQ and the free energy of ATP hydrolysis ΔG which takes into consideration the present calculation o nucleotides and phosphate:

$$\Delta Q = -\Delta G - W = \ln \frac{k_{12}k_{23}k_{31}}{k_{21}k_{32}k_{31}} = \ln \left(\frac{[ATP][ADP_0][P_{i,0}]}{[ATP_0][ADP][P_{i,0}]} e^{-W} \right), \tag{16}$$

where $W = F \cdot \Delta d$ is the mechanical work done per forward cross-bridge cycle completion. For a forward cycle completion, a power-stroke against an effectively sensed force F has to be overcome along an effective step size Δd.

2.2 Passive element with linear elasticity

In the experiment, the force P of the muscle fiber is measured. We assume, that this force distributes equally over all attached cross-bridges, so that the same load F is experienced by all attached cross-bridges:

$$F = \frac{P}{N(x_1 + x_2)}. \tag{17}$$

The force arises from stretching the muscle fiber to a length $L(t)$, which is longer than the length $L_{mol}(t)$. L_{mol} is the length which the molecular contractile apparatus would attain in its current configuration without external stretch. t denotes the time after start of the lengthening or shortening ramp. The force is connected to the difference between actual length L and molecular configuration length L_{mol} by an elastic modulus $\overline{C}$, which is assumed to be constant over all lengths of the fiber:

$$P(t) = \overline{C}(L(t) - L_{mol}(t)). \tag{18}$$

When we combine the equations (17) and (18) we get

$$F = C\frac{L(t) - L_{mol}(t)}{x_1 + x_2}, \tag{19}$$

with the effective elastic modulus $C = \overline{C}/N$ experienced by a single cross-bridge.

In a ramp experiment, $L(t)$ is typically controlled externally by the operator, and is therefore given as an external condition. Differently, $L_{mol}(t)$ changes dependent on the dynamic behavior of the cross-bridge population. The following differential equation

$$\frac{d}{dt}L_{mol} = N\left[\Delta d(k_{21}x_2 - k_{12}x_1) + \Delta d_{rip}k^0_{rip}\exp(F/\tilde{C}_{rip})x_1)\right] \tag{20}$$

describes how the rate of change of L_{mol} depends on the cross-bridge cycle transition rates. This expression incorporates the effective myosin step size Δd and the relaxation distance Δd_{rip} from a single reverse stretched pre-power-stroke cross-bridge being "ripped" off.

3 Numerical evaluation

3.1 Simulation of force curves

To implement our experiments in silico, we mimick the ramp shortening and stretching. First, we initialize the simulation at a start time $T_{wait} < 0$, whose absolute value determines how long the fiber is held isometrically at $L = 0$ before the ramp starts. This waiting time allows the fiber to contract close to the isometric force maximum, accordingly we used the value at $t = 0$ as the maximal isometric force $P_0 = P(t = 0)$. At time $t = 0$ we start a ramp length change

$$L(t) = \min(0, \max(L_{max}/(L_0 v_{ramp}), t)) \cdot v_{ramp} \cdot L_0 \tag{21}$$

which changes the fiber length $L(t)$ by a maximal length change L_{max} at a constant ramp velocity v_{ramp}. v_{ramp} is in units of $[L_0]$, which we have to choose as a model parameter. Note that $L(t)$ does not refer to the actual length of the fiber, but to the length change from the isometric length.

We evaluated our model with the MatLab ode15s adaptive time step size integrator for stiff ODEs, see Figures 2 and S2. Regular ODE integrators proved inefficient due to the rapid changes in force right after beginning of the ramp shortening.

3.2 Detection of critical points

P1 was detected as a dominant peak in the curvature $Curv$ of the force trace, P2 was detected as a characteristic transition in $log_{10}(Curv)$ from a curved decay to a linear decay, see Figure S3. The transition of a curved decay to a linear decay was detected as the first high peak in the second derivative of $log_{10}(Curv)$ with a negative value of the first derivative of $log_{10}(Curv)$.

Model parameter	Meaning	General model	Shortening model	Unit
s	Forward/backward partitioning of force dependence	0.5	0.5	–
Δd_P	Effective step size for power-stroke	50	100	–
C	Effective elastic modulus	100	20	–
L_0	Ramp length per unit time	0.8	7	–
k_P^0	Power-stroke zeroth order rate constant	1	1	$1/t_0$
k_{det}^0	Detachment zeroth order rate constant	0.0025	0.0025	$1/t_0$
k_{att}^0	Attachment zeroth order rate constant	0.025	0.025	$1/t_0$
$[P_0]$	Equilibrium concentration of phosphate	50	50	μM
K_{att}	Affinity of myosin head for binding site	8	8	–
$\tilde{c}_{rip}$	"Ripping" stretch dependence parameter	0.25	0	–
Δd_s	Step size for one ripped off myosin	80	0	–
k_{rip}^0	"Ripping" zeroth order rate constant	0.0025	0	$1/t_0$
**Concentration**	**Meaning**	**General model**	**Shortening model**	**Unit**
$[ATP]$	ATP concentration	500	500	μM
$[ADP]$	ADP concentration	5	5	μM
$[P_i]$	P_i concentration	5	5	μM

Tab. 1: **Free model parameters and conditions:** Parameters and conditions as determined by adjustment to experimental data. Nucleotide concentrations have been assigned assumed values. General model parameters include forced cross-bridge ripping and are used in the model appendix for shortening and lengthening response simulation. The shortening model parameters do not incorporate cross-bridge ripping, and are used for all other simulations and apply for all model results referenced in the main text.

3.3 Blebbistatin inhibition

To predict the effects of blebbistatin inhibition, we need to incorporate the effects of a reduced binding energy for the closing of the myosin binding cleft on actin. We set the binding energy reduction to be $\Delta E = 0.35$. As a result of the reduced binding energy, the free energy level of the tight bound state x_2 is increased. Rate constants into that state get lowered, rate constants from that state increase:

$$k'_{23} = k_{23}e^{\Delta E}, k'_{32} = k_{32}e^{-\Delta E}, k'_{12} = k_{12}e^{-\Delta E}, k'_{32} = k_{32}e^{\Delta E} \tag{22}$$

where the prime marks the rate constants for reduced binding energy. For details see main text and Figure 12, for predicted changes to the critical points see Figure 11. Another possible mechanism of blebbistatin inhibition is a lowering of the power-stroke zeroth order rate constant k_P^0. This change predicts the blebbistatin effect on the critical points accurately, see Figure S4, but a reduction of isometric maximal force P_0 is only predicted for changes in ΔE, not changes in k_P, see equation (36).

4 General conclusions from a simple model

4.1 Simplified generic model

To understand how the mechanism of blebbistatin inhibition can be properly included in our model, we will simplify it to the necessary elements:

$$\dot{x_1} = -(k_0 e^{(1-s)\Delta Q_P} + 1)x_1 + k_0 e^{-s\Delta Q_P} x_2 + K_{att} x_3 \tag{23}$$

$$\dot{x_2} = +(k_0 e^{(1-s)\Delta Q_P})x_1 - (k_0 e^{-s\Delta Q_P} + e^{\Delta Q_D})x_2 + K_{att} x_3, \tag{24}$$

where we omitted the differential equation for $x_3 = 1 - x_2 - x_1$. k_0 is the zeroth order power-stroke transition rate, all other zeroth order rates have been set equal to 1. $\Delta Q_P = -\Delta G_P - W$ is the free energy dissipation to heat during the forward power-stroke transition, $\Delta G_P < 0$ is the chemical free energy released in this transition and W is the mechanical work exerted during the power-stroke. ΔQ_D is the free energy dissipated to heat during the detachment of myosin from actin. In skeletal muscle myosin it can be equated with the free energy released in this step by ATP binding and ADP release $\Delta Q_D \approx -\Delta G_D = \text{const.}$ s is a partitioning parameter that describes the detailed load-dependence of the forward and the backward power-stroke transition.

4.2 Partitioning of bound cross-bridges at maximal isometric contraction

In a steady state $\dot{x}_1 = \dot{x}_2 = 0$ must hold. Thus, we set (23) and (24) equal to 0, and subtract these expressions. Considering further that at maximal isometric contraction

$$W = -\Delta G = -\Delta G_P - \Delta G_D \tag{25}$$

we get

$$\frac{x_1}{x_2} = \frac{e^{\Delta Q_D} + 2k_0 e^{s\Delta Q_D}}{1 + 2k_0 e^{(s-1)\Delta Q_D}}, \tag{26}$$

which describes the ratio of cross-bridges in the pre-power-stroke state over cross-bridges in the post-power-stroke state. In skeletal muscle myosin, the detachment of cross-bridges after the power-stroke is very rapid[4], which is indicative of a high free energy release

during this step: $\Delta G_D \ll 0$. Applying this condition to (26) shows $x_1/x_2 \gg 1 \Leftrightarrow x_1 \gg x_2$. Thus, at maximal isometric contraction we would expect most bound cross-bridges to be in the pre-power-stroke state, as is observed in simulations of the full model, too. Differentiation of (26) with respect to k_0 yields

$$\frac{d}{dk_0}\left(\frac{x_1}{x_2}\right) = 0, \tag{27}$$

so the zeroth order transition rate of the power-stroke does not affect the pre-post partitioning of bound cross-bridges at maximal isometric contraction.

Effect of strain-sensitive ADP release: As discussed in a review by Nyitrai and Geeves[4], strain sensitivity of ADP release in skeletal muscle myosin has not been proven directly yet, but should likely be the case. Our model does not yet include strain sensitivity of ADP release. Let us , in our simplified generic model, estimate the expected effect of stress-sensitive ADP release on the cross-bridge partitioning at maximal isometric force. Let us again take (26) and for simplicity assume $s = 1$ and $k_0 = 0.5$ without assuming $\Delta G_D = \text{const.}$:

$$\frac{x_1}{x_2} = \frac{1}{2}\left[e^{\Delta Q_D} + e^{-\Delta Q_P}\right]. \tag{28}$$

At the maximal isometric force $\Delta Q_D + \Delta Q_P = 0$ must hold in our model. In the case of not load-sensitive ADP release $\Delta Q_D = \text{const.}$ and we get $\Delta Q_P = -\Delta Q_P$, and therefore also $x_1/x_2 = e^{\Delta Q_D}$. Assuming $\Delta Q_D \gg 0$ (see [4]) we recover the case $x_1 \gg x_2$ discussed above.

Now we want to introduce a load-sensitive ADP release step instead, as hypothesized by Nyitraiy and Geeves[4]. To get an estimate of the effect we assume that the ADP release-associated heat release ΔQ_D is not constant, but $\Delta Q_P = \Delta Q_D$ instead. Then, for $\Delta Q_P + \Delta Q_D = 0$ to be fulfilled, $\Delta Q_P = \Delta Q_D = 0$ must be. With this, from (28) we get

$$x_1 = x_2. \tag{29}$$

This ad-hoc investigation indicates that stress-sensitive ADP release could actually alter the $x_1 \gg x_2$ ratio we found for no stress-sensitivity of ADP release more towards x_1 and x_2 to be of the same order of magnitude.

4.3 Maximal isometric force P_0

Now, we want to understand how the reduction of isometric maximal force P_0 by blebbistatin inhibition can be explained in our simplified model. Blebbistatin interferes with the tight binding of myosin to actin by placing itself at the interface between myosin's actin binding cleft and the actin binding site[6]. It seems reasonable that this would lead to a reduction of the binding energy associated with the tight bound state, see Figure 12 in main text. We include this reduction of binding energy ΔE into our simplified model in the following manner:

$$\dot{x_1} = -\left(k_0 e^{(1-s)\Delta Q_P - \Delta E} + 1\right)x_1 + k_0 e^{-s\Delta Q_P + \Delta E} x_2 + K_{att} x_3 \tag{30}$$

$$\begin{aligned}\dot{x_2} = &+ \left(k_0 e^{(1-s)\Delta Q_P - \Delta E}\right)x_1 - \left(k_0 e^{-s\Delta Q_P + \Delta E} + e^{\Delta Q_D + \Delta E}\right)x_2 \\ &+ K_{att} e^{-\Delta E} x_3,\end{aligned} \tag{31}$$

see also figure 12 in the main text. At maximal isometric contraction, the free energy released during one cross-bridge cycle $-\Delta G$ and the mechanical work exerted during one

cross-bridge cycle W are equal. In consequence, a detailed equilibrium is established in the three state kinetic cycle[1]. We can use the detailed equilibrium condition to express the bound cross-bridges populations in x_1 and x_2 in terms of the non-bound cross-bridge population x_3:

$$x_1 = K_{att} x_3,\ x_2 = K_{att} e^{-\Delta E - \Delta Q_D} x_3. \tag{32}$$

We substitute into $1 = x_1 + x_2 + x_3$ and get

$$x_3 = \left[1 + K_{att}\left(1 + e^{-\Delta E - \Delta Q_D}\right)\right]^{-1}. \tag{33}$$

We can now determine the fraction p_{bound} of cross-bridges that are bound:

$$p_{bound} = 1 - x_3 = \frac{K_{att}\left(1 + e^{-\Delta E - \Delta Q_D}\right)}{1 + K_{att}\left(1 + e^{-\Delta E - \Delta Q_D}\right)}. \tag{34}$$

When we assume a number N of effectively cycling cross-bridges, we can calculate the maximum isometric force P_0 which the bound cross-bridges $N_{bound} = p_{bound} N$ can sustain using $W = \Delta d P_0 / N_{bound} = -\Delta G$:

$$P_0 = -\Delta G \frac{N p_{bound}}{\Delta d}, \tag{35}$$

where Δd is an effective myosin step size. The expression $-\Delta G N / \Delta d$ is constant, so we can state the following proportionality:

$$P_0 \propto p_{bound} = \frac{K_{att}\left(1 + e^{-\Delta E - \Delta Q_D}.\right)}{1 + K_{att}\left(1 + e^{-\Delta E - \Delta Q_D}\right)} \tag{36}$$

Thus, for increasing $\Delta E \geq 0$ the maximal isometric force decreases monotonously. This is in accordance with the experimentally observed reduction in maximal isometric force P_0 with increasing blebbistatin concentrations. Also, it is clear that P_0 is not affected by changes in k_0, the zeroth order rate constant of the power-stroke transition, see (27). In consequence, a reduction ΔE in the binding energy of the tight binding of the myosin S1 actin binding cleft to actin is in our model is in accordance with the observed blebbistatin inhibition in our experiments, while a reduction in k_0 is not. This means a $\Delta E > 0$ is a necessary effect of blebbistatin inhibition, while a reduction of k_0 can additionally take place. When only the effect of a k_o reduction on the critical points along is viewed, the blebbistatin inhibition effect seems to be well predicted, see Figure S4. However, we showed in (27) that the P_0 reduction for increasing blebbistatin concentration is not explicable by a reduction of k_0.

References

[1] TL Hill. *Free energy transduction and biochemical cycle kinetics.* Dover publications, 2004.

[2] JM Laakso, JH Lewis, H Shuman, and EM Ostap. Myosin i can act as a molecular force sensor. *Science*, 321:133–136, 2008.

[3] DL Nelson and MM Cox. *Lehninger, Principles of Biochemistry.* 2005.

[4] M Nyitraiy and MA Geeves. Adenosine diphosphate and strain sensitivity in myosin motors. *Phil Trans R Soc B*, 359:1867–1877, 2004.

[5] GJ Pinniger, KW Ranatunga, and GW Offer. Crossbridge and non-crossbridge contributions to tension in lengthening rat muscle: force-induced reversal of the power stroke. *J Physiol*, 573(3):627–643, 2006.

[6] B Ramamurthy, CM Yengo, AF Straight, TJ Mitchison, and HL Sweeney. Kinetic mechanism of blebbistatin inhibition of nonmuscle myosin. *Biochem*, 43:14832–14839, 2004.

[7] H Roots, GW Offer, and KW Ranatunga. Comparison of the tension responses to ramp shortening and lengthening in intact mammalian muscle fibres: crossbridge and non-crossbridge contributions. *J Muscle Res Cell Motil*, 28:123–139, 2007.

[8] C Veigel, JE Molloy, S Schmitz, and J Kendrick-Jones. Load-dependent kinetics of force productiuon by smooth muscle myosin measured with optical tweezers. *Nature Cell Biol*, 5(11):980–986, 2003.
